# Blood pressure lowering effects of β‐blockers as add‐on or combination therapy: A meta‐analysis of randomized controlled trials

**DOI:** 10.1111/jch.14616

**Published:** 2023-02-08

**Authors:** Qian‐Hui Guo, Zhi‐Ming Zhu, Ying‐Qing Feng, Jin‐Xiu Lin, Ji‐Guang Wang

**Affiliations:** ^1^ Department of Cardiovascular Medicine State Key Laboratory of Medical Geonomics Shanghai Key Laboratory of Hypertension Center for Epidemiological Studies and Clinical Trials The Shanghai Institute of Hypertension National Research Center for Translational Medicine at Shanghai Ruijin Hospital Shanghai Jiaotong University School of Medicine Shanghai China; ^2^ Chongqing Institute of Hypertension Department of Hypertension and Endocrinology Daping Hospital Army Medical University Chongqing China; ^3^ Department of Cardiology Guangdong Cardiovascular Institute Guangdong Provincial People's Hospital Guangdong Academy of Medical Sciences Guangzhou China; ^4^ Cardiovascular Department The First Affiliated Hospital Fujian Medical University Fujian Institute of Hypertension Fuzhou China

**Keywords:** beta‐blocker, combination therapy, efficacy, hypertension, non‐atenolol

## Abstract

The authors performed a meta‐analysis to assess the efficacy of non‐atenolol β‐blockers as add‐on to monotherapy or as a component of combination antihypertensive therapy in patients with hypertension. The authors searched and identified relevant randomized controlled trials from PubMed until November 2021. Studies comparing blood pressure lowering effects of β‐blockers with diuretics, calcium channel blockers (CCBs), angiotensin‐converting enzyme inhibitors (ACEIs), or angiotensin receptor blockers (ARBs) were included. The analysis included 20 studies with 5544 participants. β‐blockers add‐on to monotherapy significantly reduced systolic and diastolic blood pressure as compared with non‐β‐blocker monotherapy (weighted mean difference in mm Hg [95% confidence interval]: −4.1 [−6.0, −2.2] and −3.7 [−4.6, −2.8], respectively). These results were consistent across the comparisons with diuretics (systolic pressure, −10.2 [−14.2, −6.2]; diastolic pressure, −5.4 [−8.2, −2.6]), CCBs (systolic pressure, −4.1 [−7.1, −1.0]; diastolic pressure, −2.8 [−4.1, −1.5]), and ACEIs/ARBs (systolic pressure, −2.9 [−4.3, −1.5]; diastolic pressure, −4.2 [−5.0, −3.4]). There was no significant difference in blood pressure lowering effects between combinations with and without a β‐blocker (systolic pressure, −1.3 mm Hg [−5.8, 3.2]; diastolic pressure, −.3 mm Hg [−2.7, 2.1]). Metoprolol add‐on or combination therapy had a significantly greater blood pressure reduction than non‐β‐blocker therapy (systolic pressure, −3.6 mm Hg [−5.9, −1.3]; diastolic pressure, −2.1 mm Hg [−3.5, −.7]). In conclusion, non‐atenolol β‐blockers are effective in lowering blood pressure as add‐on to monotherapy or as a component of combination antihypertensive therapy. In line with the current hypertension guideline recommendations, β‐blockers can and should be used in combination with other antihypertensive drugs.

## 1 INTRODUCTION

Current guidelines for the management of hypertension differ for the use of β‐blockers, despite the same clinical evidence. The Chinese Hypertension League (CHL) guidelines recommend β‐blockers as one of the first‐line antihypertensive drugs,^1^ whereas the European Society of Cardiology (ESC)/European Society of Hypertension (ESH),^2^ and the International Society of Hypertension (ISH)^3^ guidelines recommend the use of β‐blockers at any treatment step for patients with cardiovascular diseases such as myocardial infarction, heart failure, angina, or atrial fibrillation. The American Heart Association (AHA)/American College of Cardiology (ACC)^4^ and the Japanese Society of Hypertension (JSH)^5^ guidelines recommend β‐blockers for resistant hypertension and for patients with cardiovascular diseases. In consideration of the high prevalence of resistant hypertension and cardiovascular diseases, β‐blockers are often indispensable as a component of combination therapy for blood pressure control and for cardiovascular prevention and protection. However, the reluctance to use β‐blockers as part of the first‐line therapy for uncomplicated hypertension is influenced by studies with atenolol, which showed inferior effects in comparison with other classes of antihypertensive drugs.^6^, ^7^, ^8^ Indeed, in the LIFE (Losartan Intervention For Endpoint Reduction in Hypertension)^6^ and ASCOT‐BPLA (the blood pressure‐lowering arm of the Anglo‐Scandinavian Cardiac Outcomes Trial)^7^ trials with an antihypertensive regimen based on an angiotensin‐receptor blocker (ARB) and calcium‐channel blocker (CCB) as the comparator, respectively, atenolol‐based antihypertensive regimen was less efficacious in reducing blood pressure as well as the risk of cardiovascular events.

There is emerging clinical trial evidence that antihypertensive therapy with a β‐blocker other than atenolol may be particularly efficacious in blood pressure control.^9^, ^10^, ^11^ Therefore, we performed a systematic review and meta‐analysis of randomized controlled trials to assess the effects of β‐blockers, other than atenolol, as a component of antihypertensive therapy on systolic and diastolic blood pressure in patients with hypertension.

## 2 METHODS

### 2.1 Search strategy and selection criteria

We performed systematic review of randomized controlled trials with a parallel‐group design that compared blood pressure lowering effects of non‐atenolol β‐blockers as add‐on to monotherapy or as a component of combination antihypertensive therapy in patients with hypertension. We searched MEDLINE (PubMed) databases from inception to 28 November 2021. The complete search strategy is provided in Supplementary Appendix A.

Other components of combination therapy included diuretics, CCBs, angiotensin‐converting enzyme inhibitors (ACEIs), and ARBs. Among β‐blockers, atenolol was excluded, and metoprolol, bisoprolol, acebutolol, esmolol, carvedilol, labetalol, arotinolol, bevantolol, celiprolol, nebivolol, and bucindolol were included in the search. There was no restriction in terms of study duration or type of blood pressure measurement device; however, studies with a sample size smaller than 50 participants were excluded.

### 2.2 Data extraction and synthesis

**FIGURE 1:**
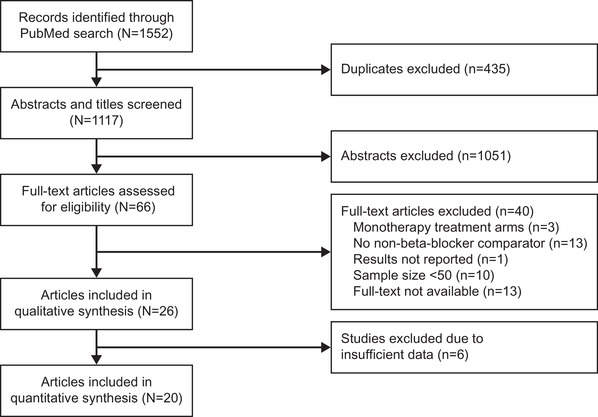
Flow diagram of selection procedure for included studies.

Studies retrieved from the MEDLINE database search were first assessed for relevance through screening of titles and abstracts. The full texts of relevant studies were then assessed for eligibility according to the inclusion and exclusion criteria set for this meta‐analysis. Prespecified data were extracted from each of the included studies by one researcher using a standardized Excel data extraction sheet, and independently reviewed by two researchers. The prespecified data extracted for each eligible study included study design, intervention characteristics, baseline characteristics of interest, and study outcomes. Any disagreements during data extraction were resolved by consensus (Figure 1).

### 2.3 Outcome measures

The outcomes of interest for the present meta‐analysis included change from baseline in clinic systolic and diastolic blood pressure and heart rate and the proportion of patients with blood pressure response.

### 2.4 Assessment of selection bias

Selection bias in eligible trials was assessed by the Cochrane collaboration's tool (version 2.0); further details are provided in Supplementary Appendices B and C. Selection bias was concurrently examined by the two data reviewers for the randomization process, deviation from intended intervention, missing outcome data, measurement of the outcome, and selection of the reported results. The overall bias for each trial was categorized as “low risk”, “some concerns”, and “high risk”.

### 2.5 Statistical analyses

All statistical analyses (pooled analyses, funnel plot, sensitivity analysis, and Harbord/Egger's test) were performed using the Stata software (version 15.0). Data were extracted using a standardized data form. The relative risks or weighted mean difference (WMD) and 95% confidence intervals (95% CIs) were calculated for each outcome using a fixed‐effects (Mantel–Haenszel method, inverse variance method) and random‐effects model, respectively, in the absence and presence of heterogeneity. All tests were two‐sided and *P* ≤ .05 was considered statistically significant. For all the outcomes of interest, subgroup analyses were performed according to therapeutic regimen.

Statistical heterogeneity was assessed using the I^2^ statistic and chi‐squared test. I^2^ statistic with values < 25, 25–50, and > 50% indicated low, moderate, and high heterogeneity, respectively. The chi‐squared test was used as inferential statistics of heterogeneity with a significance level at *P* < .1. Potential publication bias was evaluated using the funnel plot and the Harbord or Egger's test. To evaluate the influence of each study on the overall estimate, sensitivity analysis was performed in which the meta‐analysis was re‐estimated by omitting each study in turn. An individual study was suspected of excessive influence if the point estimate of its omitted analysis laid outside the CI of the combined analysis, or its omitted analysis estimate differed in significance relative to the combined analysis.

## 3 RESULTS

### 3.1 Characteristics of the included studies

Of the 1117 titles and abstracts identified in the initial search of the database, a total of 66 studies were shortlisted for full‐text review; of these, 26 papers met the inclusion criteria and reported at least one outcome of interest.^12^, ^13^, ^14^, ^15^, ^16^, ^17^, ^18^, ^19^, ^20^, ^21^, ^22^, ^23^, ^24^, ^25^, ^26^, ^27^, ^28^, ^29^, ^30^, ^31^, ^32^, ^33^, ^34^, ^35^, ^36^, ^37^ Six papers were excluded because of insufficient data^12^, ^13^, ^14^, ^15^, ^16^, ^17^; hence, 20 papers (reporting 21 treatment comparisons) comprising 5544 patients were included in the final analysis.^18^, ^19^, ^20^, ^21^, ^22^, ^23^, ^24^, ^25^, ^26^, ^27^, ^28^, ^29^, ^30^, ^31^, ^32^, ^33^, ^34^, ^35^, ^36^, ^37^ The trials were segregated and analyzed separately based on the β‐blocker interventions as add‐on to monotherapy or as a component of combination therapy. A total of 15 papers reported effects of β‐blockers as an add‐on to monotherapy compared with a non‐β‐blocker monotherapy,^18^, ^19^, ^20^, ^21^, ^22^, ^23^, ^24^, ^25^, ^26^, ^27^, ^28^, ^29^, ^30^, ^31^, ^32^ while five papers compared blood pressure lowering effects of combination therapy with and without β‐blockers.^33^, ^34^, ^35^, ^36^, ^37^

The study selection process is described in Figure 1 and details of the included studies are summarized in Table 1. Study duration ranged from 4 to 36 weeks. Of the 20 trials included in the analysis, 15 showed low risk of selection bias for the six domains of the Cochrane Risk of Bias instrument, and five trials showed some concerns of selection bias (Supplementary Appendices B and C).

**TABLE 1:** Characteristics of the included studies

Study (first author, year)	**Intervention**	**Number of patients**	Patients (inclusion criteria)	Follow‐up (weeks)
***β‐blocker add‐on therapy versus non‐β‐blocker monotherapy***				
**β‐blocker + diuretic**				
Bichisao and coworkers, 1989^31^	Metoprolol/chlorthalidone SR (200/25 mg/day) versus chlorthalidone (25 mg/day)	545	Hypertensive outpatients, at stage I or II, with supine DBP > 95 mm Hg and < 115 mm Hg	8 weeks
Labetalol/HCTZ Multicenter Study Group, 1985^32^	Labetalol (200–800 mg/day) + HCTZ (50 mg/day) versus HCTZ (50 mg/day)	174	Adults aged 19—70 years with essential hypertension (standing DBP > 95 mm Hg)	10 weeks
Frishman and coworkers, 1994^28^	Bisoprolol (10 mg/day) + HCTZ (25 mg/day) versus HCTZ (25 mg/day)	63	Adults aged ≥21 years with stage I and stage II hypertension (DBP 95–115 mm Hg)	4 weeks
Frishman and coworkers, 1995^27^	Bisoprolol (5 mg/day) + HCTZ (6.25 mg/day) versus HCTZ (25 mg/day)	283	Adults aged ≥21 years with stage I and stage II hypertension (DBP 95–115 mm Hg)	4 weeks
Prisant and coworkers, 1998^25^	Bisoprolol/HCTZ (up to 10/6.25 mg OD) versus enalapril (up to 40 mg OD) Bisoprolol/HCTZ (up to 10/6.25 mg OD) versus amlodipine (up to 10 mg OD)	462	Adults with stage I and II hypertension (DBP 95–114 mm Hg)	12 weeks
**β‐blocker + CCB**				
Dahlöf and coworkers, 1990^30^	Metoprolol/felodipine ER (100/10 mg/day) + versus felodipine ER (10 mg/day)	107	Adults aged 20–70 years with newly diagnosed or previously treated hypertension (DBP > 95 mm Hg)	12 weeks
Wetzchewald and coworkers, 1992^29^	Metoprolol (100–200 mg OD) + felodipine (10–20 mg OD) versus felodipine (10–40 mg OD)	76	Adults with DBP ≥95 mm Hg	36 weeks
Andersson and coworkers, 1999^26^	Metoprolol/felodipine (50/5 or 100/10 mg OD) versus enalapril (10–20 mg OD)	120	Adults aged 20–70 years, with supine DBP 95–115 mm Hg	8 weeks
Waeber and coworkers, 1999^23^	Metoprolol/felodipine (50/5 to 100/10 mg/day) versus enalapril (10–20 mg/day)	642	Adults with uncomplicated essential hypertension (seated DBP 95–110 mm Hg)	12 weeks
Zannad and coworkers, 1999^24^	Metoprolol/felodipine (50/5 mg OD) versus amlodipine (5 mg OD)	245	Patients aged 30–75 years with mild‐to‐moderate uncomplicated primary hypertension and DBP of 95–115 mm Hg	6 weeks
Frishman and coworkers, 2006^22^	Metoprolol/felodipine ER (400/20 mg OD) versus felodipine ER (20 mg OD)	173	Adults aged 18–80 years with uncomplicated essential hypertension (seated DBP 95–114 mm Hg)	9 weeks
Devi and coworkers, 2011^21^	Metoprolol ER/amlodipine (50/5 mg OD) versus amlodipine (5 mg OD)	163	Adults aged 18–80 years with a SBP of 140–179 mm Hg and DBP of 90–114 mm Hg	8 weeks
**β‐blocker + ACEI/ARB**				
Deedwania and coworkers, 2013^19^	Nebivolol (5–40 mg/day) + lisinopril (10 mg/day) or losartan (50 mg/day) versus lisinopril (10 mg/day) or losartan (50 mg/day)	325	Adults aged 18–80 years with a diagnosis of primary hypertension; DBP 90–110 mm Hg if untreated, 85–105 mm Hg if taking one antihypertensive medication, or 80–95 mm Hg if taking two antihypertensive medications	12 weeks
Weiss and coworkers, 2013^20^	Nebivolol (5–40 mg/day) + lisinopril (10–20 mg/day) or losartan (100–200 mg/day) versus lisinopril (10–20 mg/day) or losartan (100–200 mg/day)	491	Adults aged 18–85 years with a diagnosis of primary hypertension; SBP 170–200 mm Hg if untreated, 155–180 mm Hg if taking one antihypertensive medication, or 140–170 mm Hg if taking two antihypertensive medications	12 weeks
Giles and coworkers, 2014^18^	Nebivolol/valsartan (10/160 mg/day for Weeks 1–4 and 20/320 mg/day for Weeks 5–8) versus valsartan (160 mg/day for Weeks 1–4 and 320 mg/day for Weeks 5–8)	1108	Men and women aged ≥18 years with stage I or II hypertension (JNC7 criteria) with a recent DBP of ≥90 mm Hg and < 110 mm Hg if receiving hypertensive treatment, or ≥95 mm Hg and < 110 mm Hg at screening if untreated	8 weeks
**β‐blocker combination therapy**				
Breithaupt‐Grögler K and coworkers, 1998^37^	Metoprolol/HCTZ (100/12.5 mg) versus verapamil SR/trandolapril (180/1 mg)	51	Adults with hypertension (DBP ≥90 and < 115 mm Hg)	24 weeks
Klein and coworkers, 1998^36^	Metoprolol/felodipine (50/5 mg OD for Weeks 1–4 and 100/10 mg OD for Weeks 5–8) versus captopril/HCTZ (25/25 mg OD for Weeks 1–4 and 50/25 mg OD for Weeks 5–8)	109	Adults aged 20—70 years with mild‐to‐moderate primary hypertension (DBP 95–115 mm Hg)	8 weeks
Pareek and coworkers, 2010^35^	Metoprolol ER/amlodipine (25/2.5 mg – 50/5 mg OD) versus losartan (25–50 mg OD) + amlodipine (2.5–5 mg OD)	148	Patients aged 18—75 years with mild‐to‐moderate hypertension (DBP 90–109 mm Hg)	12 weeks
Grassi and coworkers, 2017^34^	Nebivolol/HCTZ (5/12.5 mg OD) versus irbesartan/HCTZ (150/12.5 mg OD)	122	Elderly aged > 60 years with isolated systolic hypertension (SBP ≥140 mm Hg and DBP < 90 mm Hg)	12 weeks
Farag and coworkers, 2018^33^	Nebivolol (5 mg OD) + valsartan (160 mg OD) versus amlodipine/valsartan (10/160 mg OD)	137	Patients with essential hypertension of stage II or more severe (defined as either SBP ≥160 mm Hg or DBP ≥100 mm Hg)	12 weeks

Abbreviations: ACEI, angiotensin‐converting enzyme inhibitor; ARB, angiotensin receptor blocker; CCB, calcium channel blocker; DBP, diastolic blood pressure; ER, extended‐release; HCTZ, hydrochlorothiazide; JNC7, Seventh Report of the Joint National Committee on Prevention, Detection, Evaluation, and Treatment of High Blood Pressure; OD, once daily; SBP, systolic blood pressure, SR, slow‐release.

### 3.2 Efficacy of β‐blockers as an add‐on to antihypertensive therapy

**FIGURE 2:**
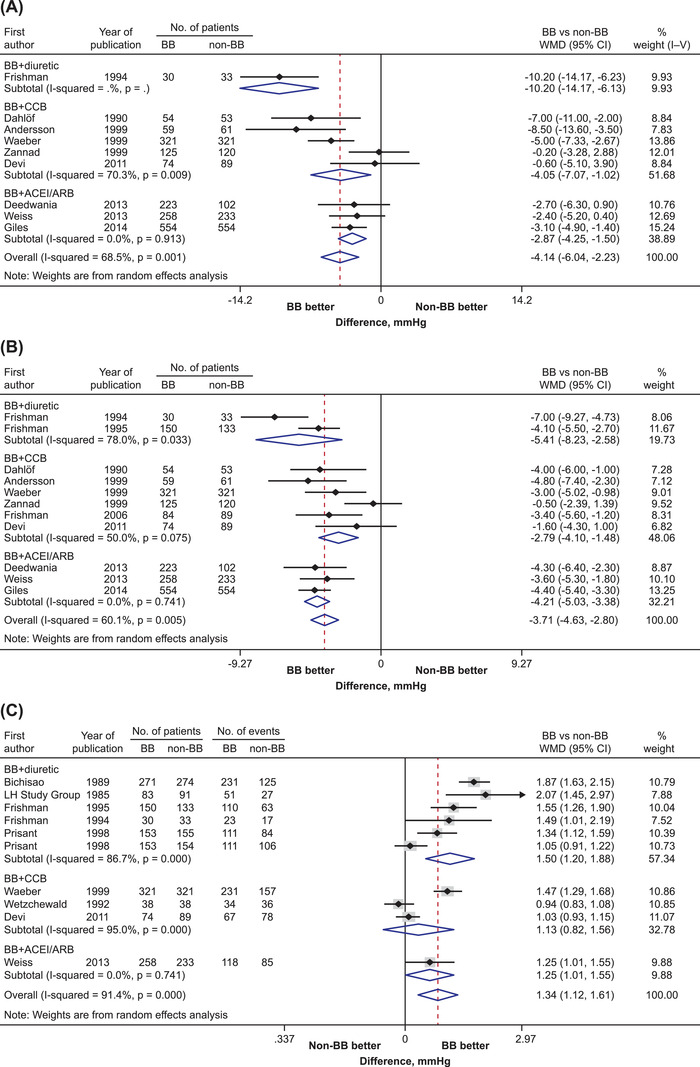
Treatment effects of β‐blocker add‐on therapy on (A) systolic and (B) diastolic blood pressure in the sitting or supine position, and (C) diastolic blood pressure response. Black symbols represent point estimate of each individual trial. Horizontal lines denote 95% CIs of each individual trial. Diamonds represent overall or subtotal pooled estimate and 95% CI of trials. ACEI, angiotensin‐converting enzyme inhibitor; ARB, angiotensin receptor blocker; BB, β‐blocker; CCB, calcium channel blocker; CI, confidence interval; WMD, weighted mean difference

Of the 15 add‐on therapy trials, nine and 11 reported changes in sitting/supine systolic and diastolic blood pressure, respectively. Overall, the β‐blocker add‐on therapy significantly reduced systolic and diastolic blood pressure more than the non‐β‐blocker monotherapy (WMD [95% CI]: −4.1 mm Hg [−6.0, −2.2] and −3.7 mm Hg [−4.6, −2.8], respectively). In the analysis according to the antihypertensive medication, systolic blood pressure reduction was significantly and consistently greater for the β‐blocker add‐on to a diuretic (WMD: −10.2 mm Hg, 95% CI: −14.2, −6.2), a CCB (WMD: −4.1 mm Hg, 95% CI: −7.1, −1.0), or an ACEI/ARB (WMD: −2.9 mm Hg, 95% CI: −4.3, −1.5; Figure 2A), compared to the respective monotherapies. Similar results were observed for diastolic blood pressure, with the corresponding WMD of −5.4 mm Hg (95% CI: −8.2, −2.6), −2.8 mm Hg (95% CI: −4.1, −1.5), and −4.2 mm Hg (95% CI: −5.0, −3.4), respectively (Figure 2B).

Ten studies reported effects on the proportion of patients who achieved a diastolic blood pressure control (< 90 mm Hg or < 95 mm Hg, or a ≥10 mm Hg reduction from baseline). Overall, the probability of patients achieving a diastolic blood pressure response was 34% higher with the β‐blocker add‐on therapy than non‐β‐blocker monotherapy (odds ratio [OR]: 1.34, 95% CI: 1.12, 1.61). In the analysis according to antihypertensive medication, the likelihood of achieving a diastolic blood pressure response was higher for the β‐blocker add‐on to a diuretic (OR: 1.50, 95% CI: 1.20, 1.88) and to an ACEI/ARB (OR: 1.25, 95% CI: 1.01, 1.55), but not to a CCB (OR: 1.13; 95% CI: .82, 1.55), compared to the respective non‐β‐blocker monotherapies (Figure 2A).

Three trials (all with metoprolol) reported treatment effects on heart rate. β‐blocker add‐on led to a greater reduction in heart rate by 6.9 bpm (95% CI: −8.6, −5.2) than non‐β‐blocker monotherapy in a fixed‐effect model (Supplementary Appendix D).

None of the included studies had excessive influence on the overall results of the meta‐analysis (Supplementary Appendix E). Funnel plots did not show any evidence of publication bias (Supplementary Appendix F).

### 3.3 Efficacy of β‐blockers as a component of combination antihypertensive therapy

**FIGURE 3:**
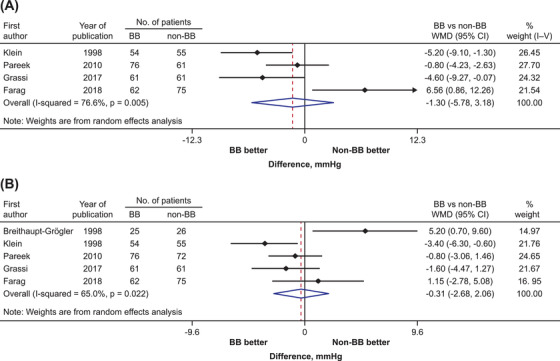
Treatment effects of β‐blocker combination therapy on (A) systolic and (B) diastolic blood pressure in the sitting or supine position. Black symbols represent point estimate of each individual trial. Horizontal lines denote 95% CIs of each individual trial. Diamonds represent overall or subtotal pooled estimate and 95% CI of trials. BB, β‐blocker; CI, confidence interval; WMD, weighted mean difference.

Five trials reported changes in sitting/supine systolic and diastolic blood pressure between combination antihypertensive therapy with and without β‐blocker as a component. There was no significant difference between β‐blocker combination and non‐β‐blocker combination therapies for the reduction in systolic and diastolic blood pressure (WMD [95% CI]: −1.3 mm Hg [−5.8, 3.2] and −.3 mm Hg [−2.7, 2.1], respectively; Figure 3A and B).

### 3.4 Subgroup analysis of metoprolol add‐on or combination antihypertensive therapy

**FIGURE 4:**
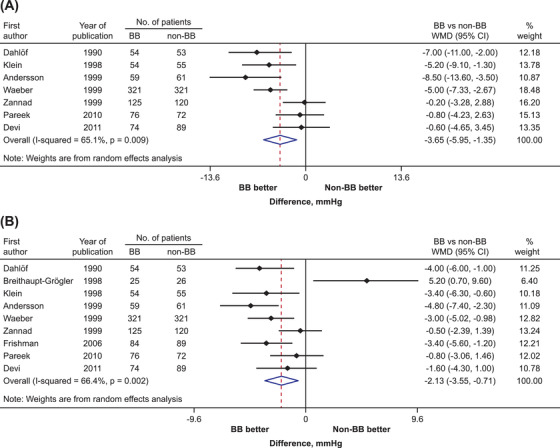
Treatment effects on (A) systolic and (B) diastolic blood pressure in the sitting or supine position in metoprolol trials. Black symbols represent point estimate of each individual trial. Horizontal lines denote 95% CIs of each individual trial. BB, β‐blocker; CI, confidence interval; WMD, weighted mean difference.

Seven and nine trials reported changes in sitting/supine systolic and diastolic blood pressure with metoprolol add‐on and combination therapy, respectively. Overall, the metoprolol add‐on or combination therapy was associated with a significantly greater reduction in systolic and diastolic blood pressure than non‐β‐blocker therapy (WMD [95% CI]: −3.6 mm Hg [−5.9, −1.3] and −2.1 mm Hg [−3.5, −.7], respectively; Figure 4A and B).

## 4 DISCUSSION

In the present meta‐analysis, β‐blocker add‐on therapy was associated with a significantly greater reduction in systolic and diastolic blood pressure compared with non‐β‐blocker monotherapy. The between‐group WMD was −4.1 and −3.7 mm Hg, respectively, consistently in favor of the β‐blocker add‐on therapy compared to various antihypertensive therapies such as diuretics, CCBs and ACEIs/ARBs. Combination therapy containing a β‐blocker was as effective as non‐β‐blocker combination therapy for blood pressure lowering effects. Similar results were observed for the analysis restricted to metoprolol trials.

This meta‐analysis investigates the blood pressure lowering effect of β‐blockers as add‐on and a component of combination antihypertensive therapy. The results of the analysis support the position that beta‐blockers can be used in combination with the other classes of antihypertensive drugs at any stage of treatment for blood pressure control, including as a first‐choice therapy. The findings of the analysis are also in line with results of previous meta‐analyses of β‐blocker therapy for the management of hypertension and other cardiovascular diseases and with the view expressed by Mancia and coworkers^38^ and Esler and coworkers^39^ in their recent reviews. After thorough review of the evidence, these authors highlighted favorable effects of β‐blockers in about 50 different clinical conditions that may coexist with hypertension. The authors also argued that downgrading β‐blockers from first‐choice therapy to use on specific conditions alone may not be justified based on the evidence that β‐blockers are as effective as other antihypertensive drugs in lowering blood pressure.^2^, ^38^, ^39^ However, it is noteworthy that β‐blockers are a heterogenous class of drugs with differences in physiochemical properties and receptor selectivity, leading to variable pharmacological effects and efficacy in patients with hypertension. Previous randomized controlled trials often used propranolol or atenolol in the evaluation of the efficacy of β‐blockers for the primary prevention of cardiovascular events. Propranolol is a non‐selective β‐blocker. Atenolol is a hydrophilic β1 selective agent with a relatively short half‐life. The once‐daily dosing of atenolol was probably sub‐optimal, as indicated by significantly lower blood pressure reduction in the atenolol group than in the comparator groups in the LIFE and ASCOT‐BPLM trials.^6^, ^7^ The results of these atenolol trials probably do not apply to other β1 selective blockers such as metoprolol. Indeed, in the Metoprolol Atherosclerosis Prevention in Hypertensives (MAPHY) trial, metoprolol treatment led to a significant reduction in cardiovascular mortality and morbidity, including stroke, compared with diuretics.^40^, ^41^ Furthermore, a previous meta‐analysis has shown that β‐blockers are effective as other classes of antihypertensive drugs in preventing cardiovascular events, especially when trials with atenolol were excluded.^42^ The findings of our current study further add to the evidence in supporting the efficacy of β‐blockers in hypertension.

If used properly, such as in combination with other classes of antihypertensive drugs, β‐blockers could be particularly effective in blood pressure lowering and in cardiovascular disease prevention. The observed 3–4 mm Hg superior blood pressure lowering effect may explain the results from several recent observational studies demonstrating outcome benefits of β‐blockers in cardiovascular disease prevention.^43^, ^44^ In a real‐world evidence study of long‐term effectiveness of β‐blockers, using data from patients registered in the UK Clinical Practice Research Datalink between 2000 and 2014 (*n* = 100 066),^44^ patients receiving β‐blocker therapy (*N* = 4240) had a lower risk of mortality than those receiving other antihypertensive therapy. The risk reduction in mortality was sustained for 15 years (hazard ratio, .52; 95% CI: .27, .10), with a 2–3 year delayed effect after the combination of β‐blocker therapy.^44^ Similar findings were reported from the long‐term follow‐up (20 years) of the UKPDS study, where the risk of mortality was lower with a β‐blocker than with an ACEI in patients with diabetes mellitus and hypertension.^43^

The observed greater blood pressure lowering effect of β‐blockers add‐on to combination ACEIs/ARBs is noteworthy. Such a combination is not recommended by any current hypertension guidelines, because β‐blockers suppress renin secretion and reduce the plasma levels of angiotensin II and, therefore, perceived to be less additive in antihypertensive efficacy to ACEIs/ARBs.^45^ The additive effect of β‐blockers add‐on to ACEIs/ARBs on blood pressure reduction was indeed less than the add on to diuretics or CCBs, but still appreciable in size, at 2–4 mm Hg greater than antihypertensive therapy with non‐β‐blockers. Of note, combination therapy with ACEIs/ARBs and a β‐blocker is recommended as first‐line therapy in patients with heart failure or myocardial infarction.^46^, ^47^, ^48^, ^49^ While the majority of these patients also have hypertension, the recommendations are not solely based on the blood pressure lowering effects of β‐blockers, but rather on their cardioprotective effects. The efficacy of β‐blockers with ACEI/ARBs combination therapy in hypertension remains unexplored and should be further investigated in randomized controlled trials.

The present meta‐analysis should be interpreted within the context of its limitations. First, the analysis was based on summary statistics instead of individual patient data, hence reaching less standardization. Second, the number of trials and study patients were relatively small for some of the subgroup analyses, for example, the combination of β‐blockers and ACEIs/ARBs and the metoprolol versus non‐metoprolol subgroup analyses. The pooled estimates on the subgroup analyses require confirmation in further randomized controlled trials. In addition, the dose of the study drugs varied across studies. However, sensitivity analyses did not show any excessive influence of any individual study on the pooled estimates. Finally, the duration of the included studies was relatively short (ranged from 4 to 36 weeks). The study results cannot be extrapolated to longer‐term duration. Nonetheless, metoprolol demonstrated sustained blood pressure lowering effects in the long‐term MAPHY trial with a median follow‐up of 4.2 years.^40^, ^41^

In conclusion, this meta‐analysis showed that β‐blockers (excluding atenolol) as add‐on to several other classes of antihypertensive drugs, such as diuretics, CCBs and even ACEIs/ARBs, were efficacious in further lowering blood pressure and that combination therapy with β‐blockers is as effective as non‐β‐blocker combination therapy. Thus, in line with the current guideline recommendations, β‐blockers can and should be used in combination with other classes of antihypertensive drugs at any stage of treatment.

## AUTHOR CONTRIBUTIONS

All authors contributed to the meta‐analysis design and data analysis, and to the drafting, review and final approval of the manuscript.

## CONFLICT OF INTEREST

Qian‐Hui Guo and Ji‐Guang Wang were financially supported by grants from the National Natural Science Foundation of China (91639203, 82070435, and 82000394) and the Ministry of Science and Technology (2018YFC1704902), Beijing; the Shanghai Commissions of Science and Technology (19DZ2340200) and Health (“Three‐year Action Program of Shanghai Municipality for Strengthening the Construction of Public Health System”, GWV‐10.1‐XK05, and a special grant for “leading academics”); and the Clinical Research Program, Ruijin Hospital, Shanghai Jiao Tong University School of Medicine (grant number: 2018CR010), Shanghai, China.

Zhi‐Ming Zhu, Ying‐Qing Feng, Jin‐Xiu Lin declare no conflict of interest.

## Supporting information

Supporting Information
