# Supplementary material for: Blood pressure lowering effects of β‐blockers as add‐on or combination therapy: A meta‐analysis of randomized controlled trials
Source: J Clin Hypertens (Greenwich). 2023 Feb 8;25(3):227–37. doi: 10.1111/jch.14616 (PMC9994166; doi:10.1111/jch.14616)

**Blood pressure lowering effects of β-blockers as add-on or combination therapy: A meta-analysis of randomized controlled trials**

Qian-Hui Guo^1^, Zhi-Ming Zhu^2^, Ying-Qing Feng^3^, Jin-Xiu Lin^4^, Ji-Guang Wang^1^

**SUPPLEMENTARY APPENDIX**

[Appendix A. Search strategy. 2](#_Toc109828518)

[Appendix B. Risk of bias graph presenting each risk of bias item as percentage across all included studies. 3](#_Toc109828519)

[Appendix C. Risk of bias summary for included studies. 4](#_Toc109828520)

[Appendix D. Treatment effects of β-blocker add-on therapy on heart rate. 5](#_Toc109828521)

[Appendix E. Sensitivity analyses. 6](#_Toc109828522)

[Appendix F. Funnel plot of included studies. 12](#_Toc109828523)

## Appendix A. Search strategy.

**Database:** Medline (via PubMed)

**Timeline:** First literature search conducted in March 2020; Subsequent search for updates conducted in October 2021

| **Search** | **Query** | **Items found** |
| --- | --- | --- |
| **#1** | Search (Metoprolol OR Bisoprolol OR Acebutolol OR Esmolol OR Betaxolol OR Carvedilol OR labetalol OR arotinolol OR bevantolol OR Celiprolol OR Nebivolol OR Bucindolol) AND (benazepril OR enalapril OR perindopril OR fosinopril OR ramipril OR imidapril OR captopril OR lisinopril) AND "Hypertension"[Mesh]; Filters: Humans | 378 |
| **#2** | Search (Metoprolol OR Bisoprolol OR Acebutolol OR Esmolol OR Betaxolol OR Carvedilol OR labetalol OR arotinolol OR bevantolol OR Celiprolol OR Nebivolol OR Bucindolol) AND (valsartan OR irbesartan OR losartan OR olmesartan OR telmisartan OR allisartan OR candesartan) AND "Hypertension"[Mesh]; Filters: Humans | 133 |
| **#3** | Search (Metoprolol OR Bisoprolol OR Acebutolol OR Esmolol OR Betaxolol OR Carvedilol OR labetalol OR arotinolol OR bevantolol OR Celiprolol OR Nebivolol OR Bucindolol) AND (nifedipine OR amlodipine OR levamlodipine OR felodipine OR benidipine OR diltiazem OR lercanidipine OR nicardipine OR cilnidipine OR lacidipine) AND "Hypertension"[Mesh]; Filters: Humans | 550 |
| **#4** | Search (Metoprolol OR Bisoprolol OR Acebutolol OR Esmolol OR Betaxolol OR Carvedilol OR labetalol OR arotinolol OR bevantolol OR Celiprolol OR Nebivolol OR Bucindolol) AND (torasemide OR spironolactone OR furosemide OR indapamide OR bumetanide OR hydrochlorothiazide OR tolvaptan OR amiloride OR chlorthalidone OR bendroflumothiazide) AND "Hypertension"[Mesh]; Filters: Humans | 491 |
| **#5** | Remove duplicates | **1117** |

## Appendix B. Risk of bias graph presenting each risk of bias item as percentage across all included studies.


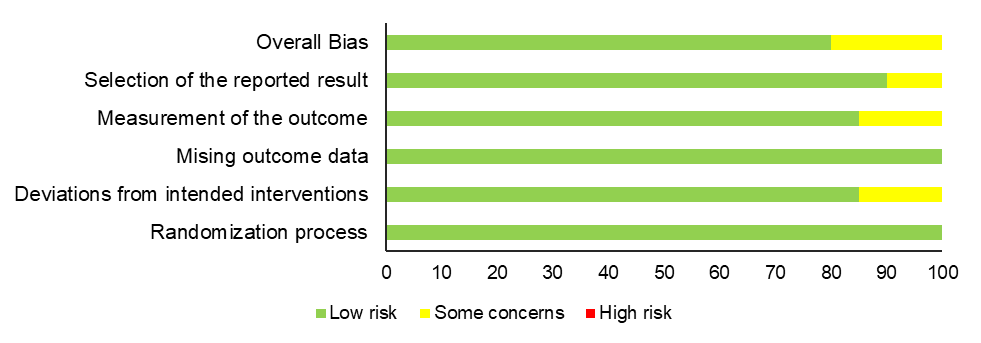


## Appendix C. Risk of bias summary for included studies.


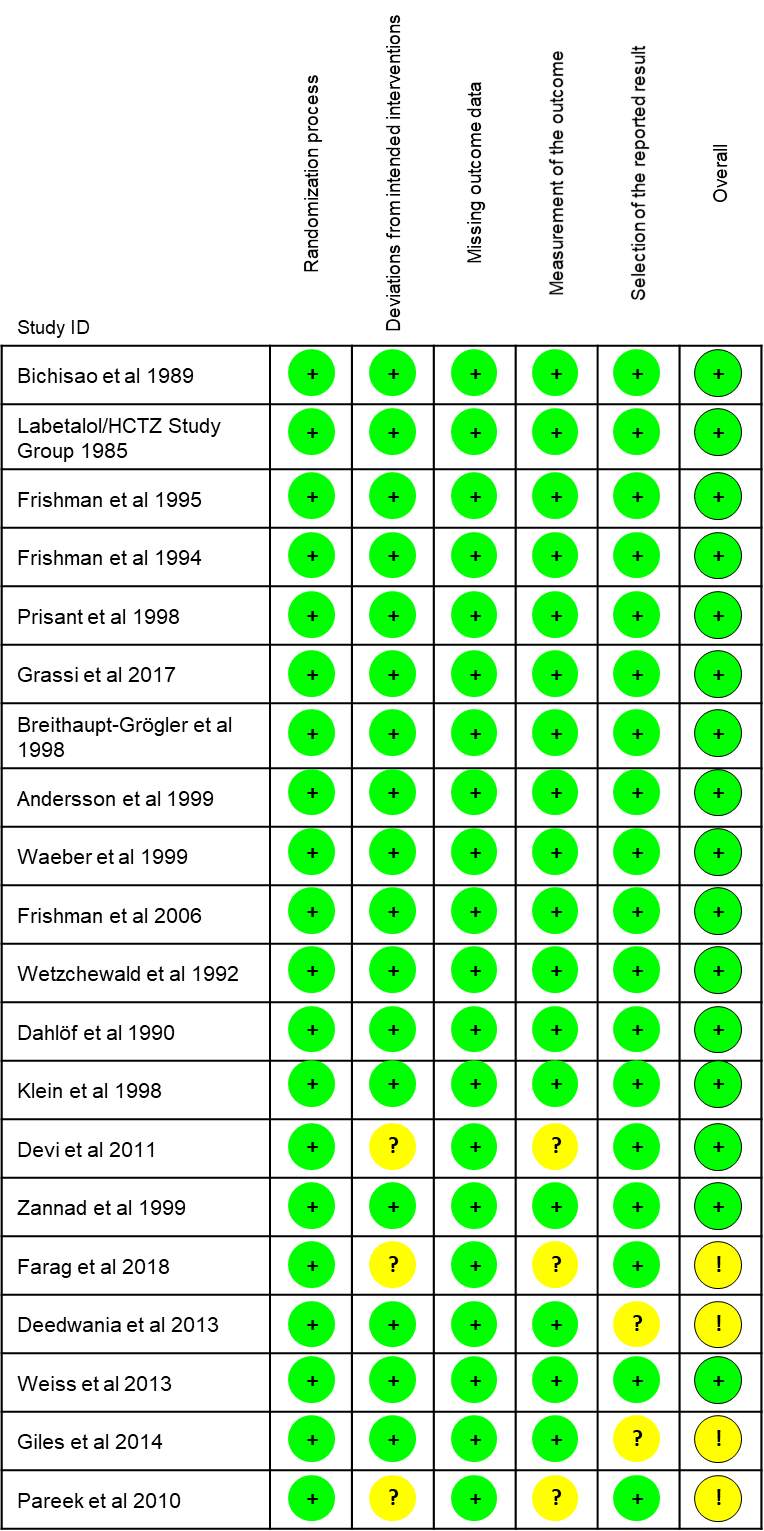


## Appendix D. Treatment effects of β-blocker add-on therapy on heart rate.

**
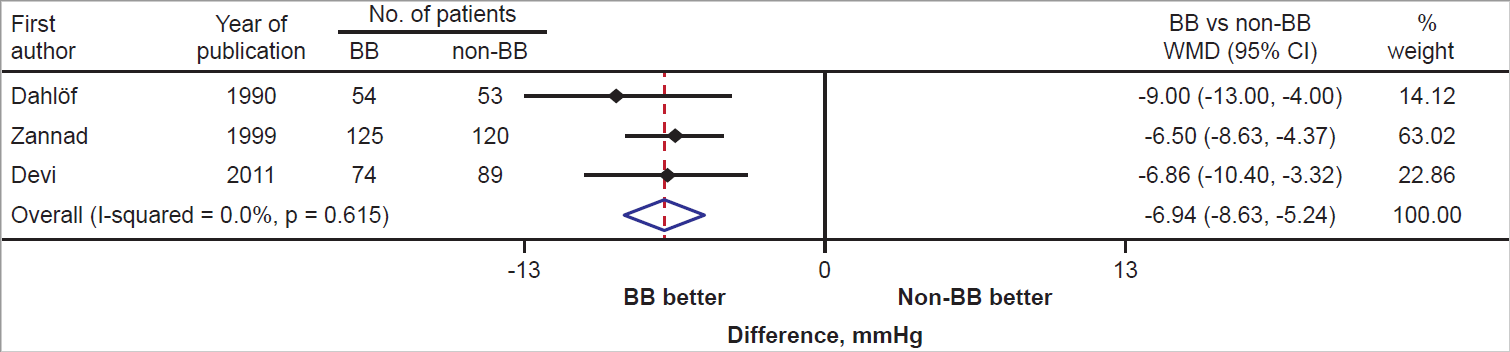
**

Black symbols represent point estimate of each individual trial. Horizontal lines denote 95% CIs of each individual trial. Diamonds represent overall or subtotal pooled estimate and 95% CI of trials.

BB, β-blocker; CI, confidence interval; WMD, weighted mean difference.

## Appendix E. Sensitivity analyses.

1. Treatment effects of β-blocker add-on therapy on SBP in the sitting or supine position

| Study omitted | Estimate | [95% Confidence Interval] | |
| --- | --- | --- | --- |
| Dahlöf, B (1990) | -3.8578739 | -5.8514147 | -1.8643332 |
| Frishman, WH (1994) | -3.397969 | -4.9973764 | -1.7985615 |
| Waeber, B (1999) | -4.6443367 | -6.5653095 | -2.7233634 |
| Zannad, F (1999) | -4.0402999 | -6.2477002 | -1.8328993 |
| Andersson, OK (1999) | -3.7557349 | -5.6689086 | -1.8425616 |
| Devi, P (2011) | -4.4808836 | -6.4807296 | -2.4810376 |
| Weiss, RJ (2013) | -4.4151034 | -6.5730243 | -2.2571824 |
| Deedwania, P (2013) | -4.3345308 | -6.4490442 | -2.2200177 |
| Giles, TD (2014) | -4.3726988 | -6.7162395 | -2.0291584 |
| Combined | -4.1353532 | -6.037153 | -2.2335534 |

1. Treatment effects of β-blocker add-on therapy on DBP in the sitting or supine position

| Study omitted | Estimate | [95% Confidence Interval] | |
| --- | --- | --- | --- |
| Frishman, WH (1995) | -3.6603189 | -4.7038622 | -2.6167755 |
| Frishman, WH (1994) | -3.4525487 | -4.2716651 | -2.6334326 |
| Andersson, OK (1999) | -3.6273055 | -4.5990825 | -2.6555283 |
| Waeber, B (1999) | -3.7821686 | -4.7728591 | -2.7914784 |
| Frishman, WH (2006) | -3.7397459 | -4.7326579 | -2.7468338 |
| Dahlöf, B (1990) | -3.6883054 | -4.6728411 | -2.7037699 |
| Devi, P (2011) | -3.8669317 | -4.7922592 | -2.9416044 |
| Zannad, F (1999) | -4.0782685 | -4.7750854 | -3.3814511 |
| Deedwania, P (2013) | -3.6535506 | -4.6512413 | -2.6558599 |
| Weiss, RJ (2013) | -3.7229719 | -4.7405477 | -2.7053962 |
| Giles, TD (2014) | -3.6111317 | -4.6647425 | -2.5575209 |
| Combined | -3.7130751 | -4.6254872 | -2.800663 |

1. Treatment effects of β-blocker add-on therapy on DBP response

| Study omitted | Estimate | [95% Conf. Interval] | |
| --- | --- | --- | --- |
| Bichisao, E (1989) | 1.2842206 | 1.091717 | 1.5106685 |
| LH Study Group (1985) | 1.2954875 | 1.0813198 | 1.5520736 |
| Frishman, WH (1995) | 1.3245158 | 1.0923184 | 1.6060722 |
| Frishman, WH (1994) | 1.3340677 | 1.1046854 | 1.6110801 |
| Prisant, LM (1998) | 1.3477054 | 1.1025113 | 1.6474296 |
| Prisant, LM (1998) | 1.3865036 | 1.135383 | 1.6931663 |
| Waeber, B (1999) | 1.3326435 | 1.088665 | 1.6312996 |
| Wetzchewald, D (1992) | 1.4006948 | 1.1771276 | 1.6667235 |
| Devi, P (2011) | 1.3887163 | 1.1539364 | 1.6712646 |
| Weiss, RJ (2013) | 1.3571469 | 1.1123892 | 1.6557583 |
| Combined | 1.3447207 | 1.1240753 | 1.6086766 |

1. Treatment effects of β-blocker add-on therapy on heart rate

| Study omitted | Estimate | [95% Confidence Interval] | |
| --- | --- | --- | --- |
| Dahlöf, B (1990) | -6.5958381 | -8.420537 | -4.7711391 |
| Devi, P (2011) | -6.9576383 | -8.8829613 | -5.0323148 |
| Zannad, F (1999) | -7.6770697 | -10.457648 | -4.8964915 |
| Combined | -6.9353155 | -8.6262866 | -5.2443445 |

1. Treatment effects on SBP in the sitting or supine position in metoprolol trials

| Study omitted | Estimate | [95% Confidence Interval] | |
| --- | --- | --- | --- |
| Dahlöf, B (1990) | -3.1841547 | -5.61935 | -.7489596 |
| Klein, G (1998) | -3.4264824 | -6.0556512 | -.79731357 |
| Zannad, F (1999) | -4.2957525 | -6.6269541 | -1.9645507 |
| Andersson, OK (1999) | -3.0544415 | -5.326828 | -.78205514 |
| Waeber, B (1999) | -3.3967748 | -6.1564112 | -.63713831 |
| Pareek, A (2010) | -4.1631436 | -6.6862388 | -1.6400486 |
| Devi, P (2011) | -4.1270895 | -6.6285653 | -1.625614 |
| Combined | -3.6508798 | -5.952634 | -1.3491255 |

1. Treatment effects on DBP in the sitting or supine position in metoprolol trials

| Study omitted | Estimate | [95% Confidence Interval] | |
| --- | --- | --- | --- |
| Dahlöf, B (1990) | -1.886779 | -3.4196522 | -.35390568 |
| Klein, G (1998) | -1.9706615 | -3.5284717 | -.41285121 |
| Breithaupt-Grögler, K (1998) | -2.5876186 | -3.6964397 | -1.4787976 |
| Andersson, OK (1999) | -1.8116516 | -3.2625918 | -.36071125 |
| Waeber, B (1999) | -1.9775932 | -3.608577 | -.34660923 |
| Zannad, F (1999) | -2.3791103 | -3.9025714 | -.85564935 |
| Frishman, WH (2006) | -1.9364663 | -3.5268445 | -.34608838 |
| Pareek, A (2010) | -2.2988064 | -3.8632185 | -.73439449 |
| Devi, P (2011) | -2.1749852 | -3.7604415 | -.58952868 |
| Combined | -2.1307916 | -3.5482298 | -.71335335 |

## Appendix F. Funnel plot of included studies.

1. Treatment effects of β-blocker add-on therapy on SBP in the sitting or supine position


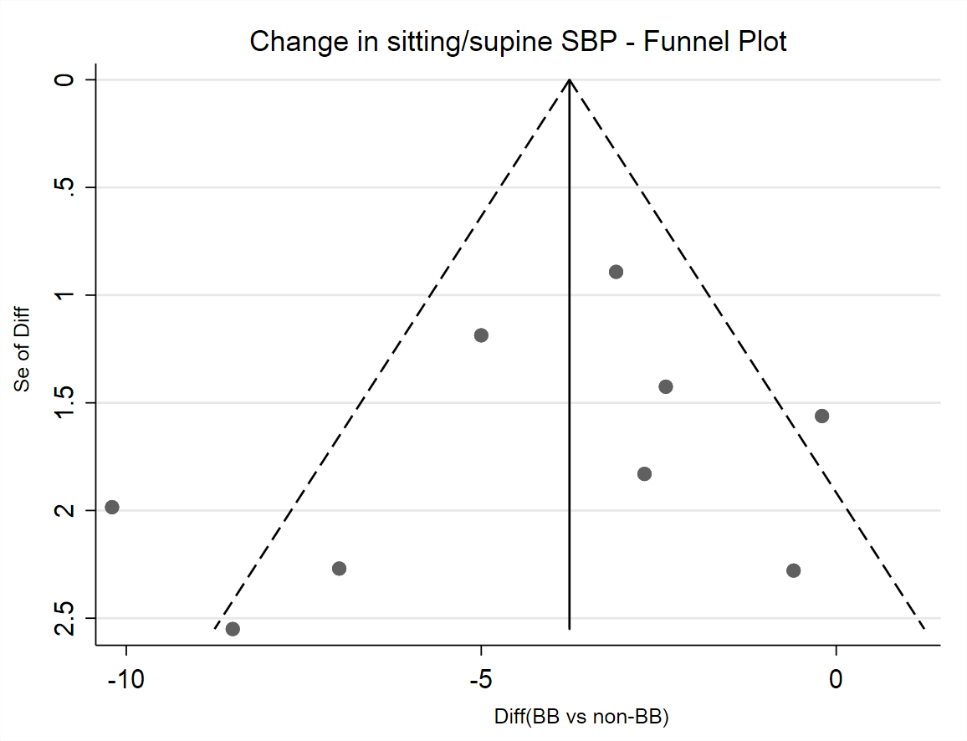


1. Treatment effects of β-blocker add-on therapy on DBP in the sitting or supine position


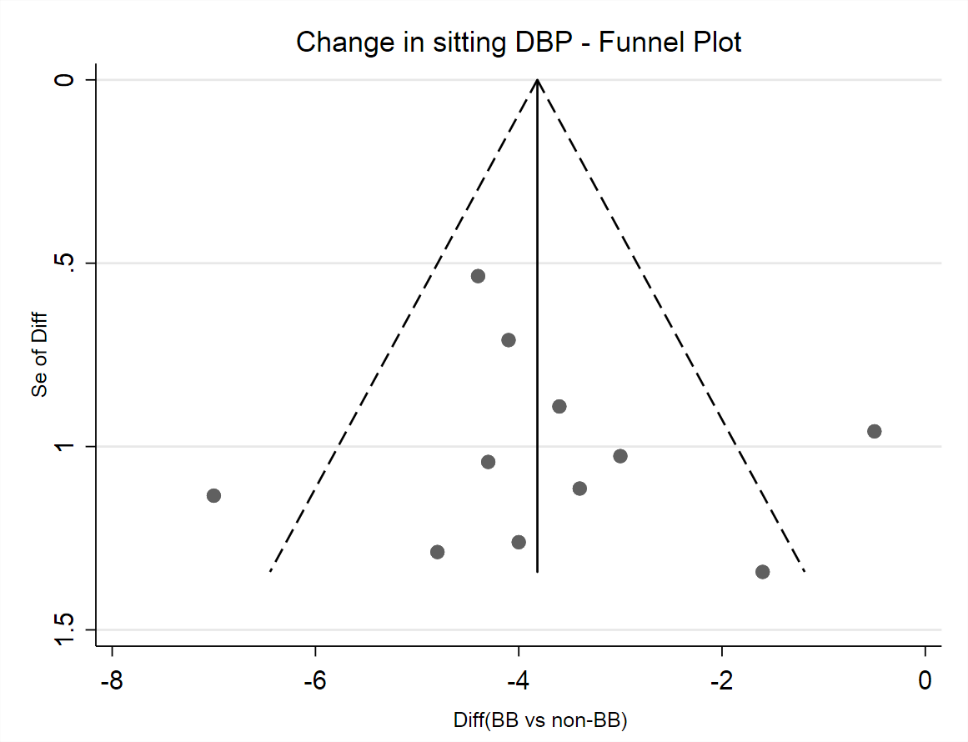


1. Treatment effects of β-blocker add-on therapy on DBP response


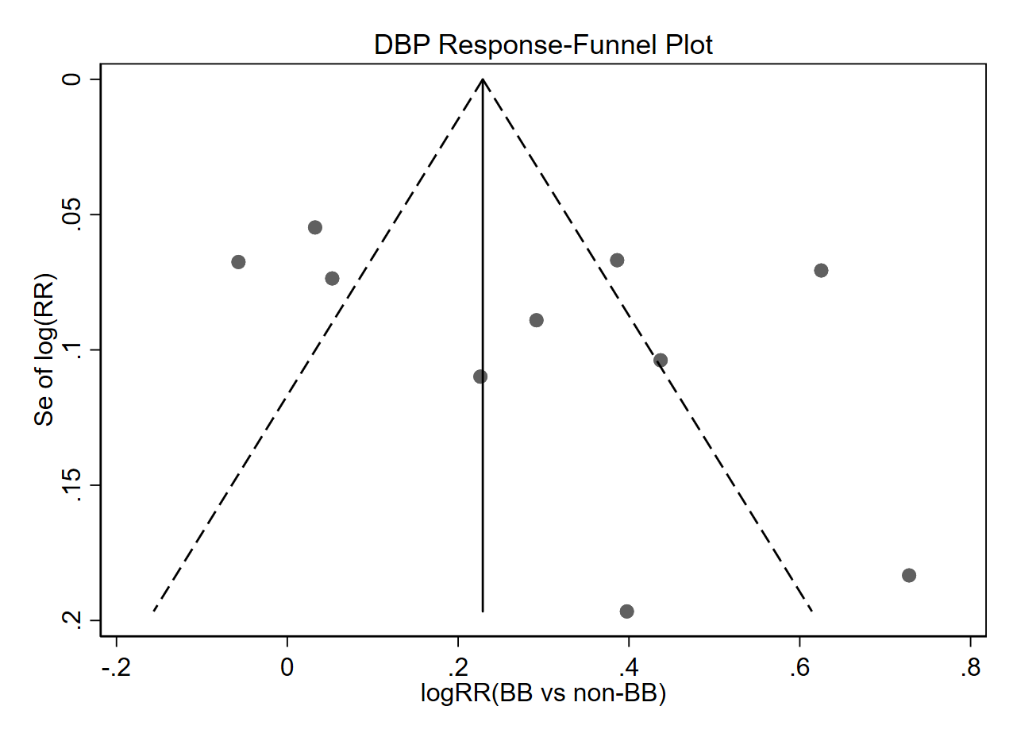


1. Treatment effects of β-blocker add-on therapy on heart rate


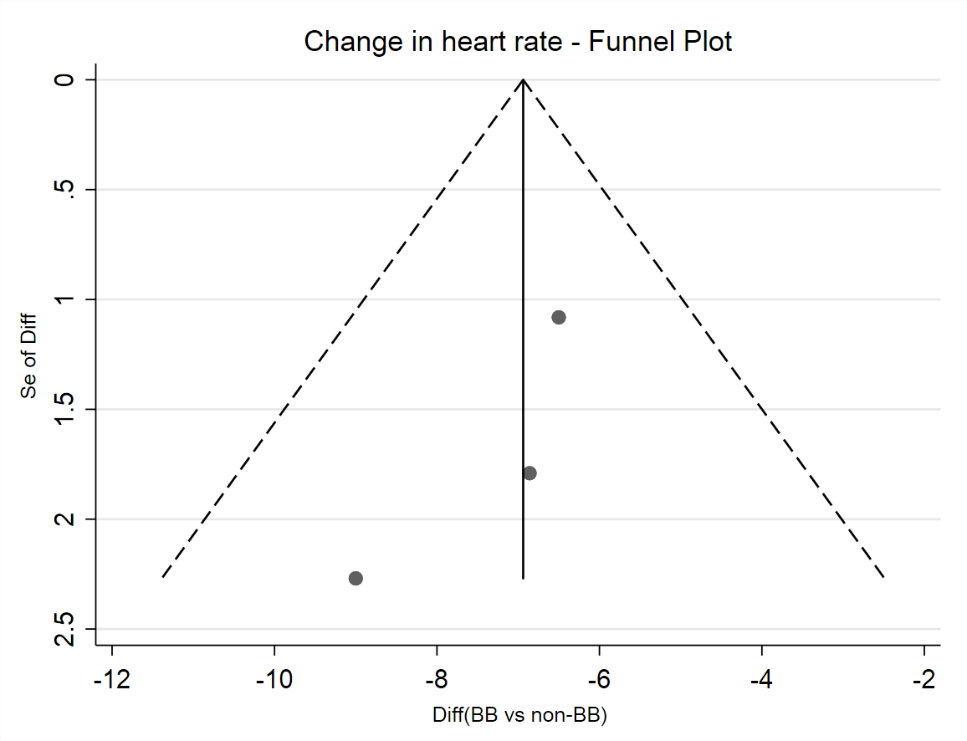


1. Treatment effects on SBP in the sitting or supine position in metoprolol trials


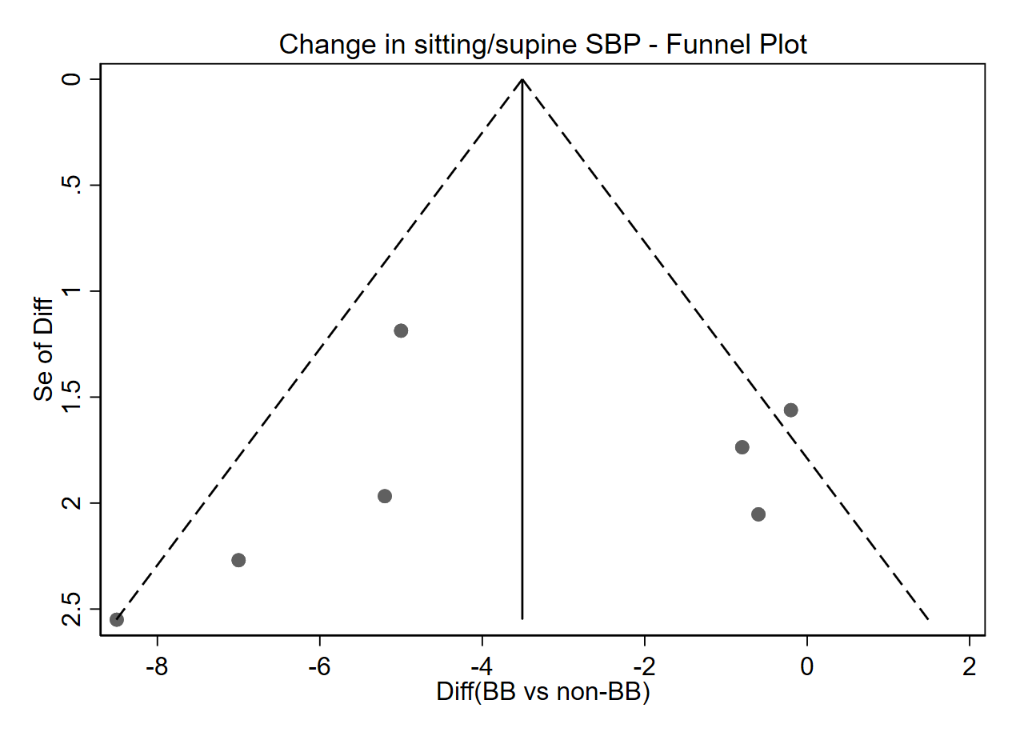


1. Treatment effects on DBP in the sitting or supine position in metoprolol trials


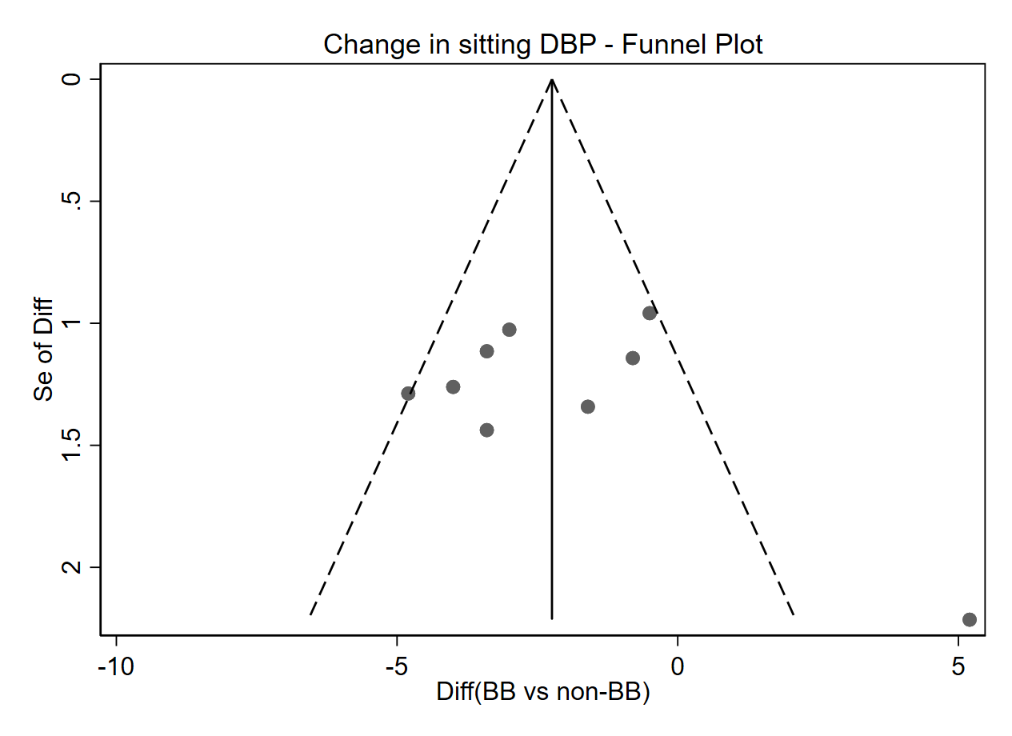

Supplement: Supplementary file 1 — Supporting Information [file JCH-25-227-s001.docx]
